# Design of Flute Music Remote Teaching System Based on Multi-Pass Scheduling Optimization

**DOI:** 10.1155/2022/1126785

**Published:** 2022-09-29

**Authors:** Zhao Jing

**Affiliations:** North University of China, Taiyuan, Shanxi 030051, China

## Abstract

With the gradual development of the Internet industry, every aspect of people's life has been affected by the Internet, playing increasingly irreplaceable functions in people's entertainment, office, and other aspects. Judging from the current development situation, the old Internet digital teaching system has many problems, such as low artificial intelligence, weak information processing ability, and lack of effective learning ability. This paper designs the flute music remote teaching system, which can realize remote music teaching and provide help in providing real-time music teaching. The music learning system includes the user's access records, the user's operation and the completion of the test data, the discussion and communication of online participation, the user's interests, specialties and operation methods, learning progress and scoring, and so on. In addition, it explores and explains all the key steps required by the current distance education model and invents a sample of the distance education model. On this basis, Internet algorithm programs will be used for all key processing functions of the system. The use of Internet algorithm programs is interactive and automated, which greatly enhances the role of the education system. This article first discusses the unique teaching and automated teaching mode of the system, which lays the cornerstone for further reforms in this field in the future.

## 1. Introduction

Due to the advent of the digital age of the Internet, a variety of Internet digital algorithm programs continue to appear. The digitalization of the Internet has brought great convenience to people in communication, entertainment, information sharing, entertainment, online shopping, and many other aspects [1]. Manager algorithm program is the field of artificial intelligence algorithm program development. The software invented as algorithm program has a certain degree of intelligence, can help people make decisions, and can independently complete certain commands such as human behavior [2]. Moreover, it has certain self-improvement skills and can reason and predict things in development [3]. With the continuous maturity of algorithm programs, the use of various fields is also increasing. In today's distance education mode, the application of algorithm programs to realize the ancient and tedious Internet digital education has become an epoch-making role in the development of distance education systems in the current era [4]. The long history of flute art began in distant European lands and has been introduced to China for nearly 150 years. In the past 100 years, Chinese flute art has undergone rapid development. The reason for the popularity of flute art at present is firstly inseparable from its beautiful tunes [5]. The treble part is subtle and elegant, the mid-range part is solid and mellow, and the bass part is thick and deep. After listening, the audience stays and does not want to leave. Then, the flute looks good and is easy to store [6]. Finally, with the exchange and penetration of various countries and fields, the Chinese people have improved their understanding of symphony, accepting and loving the “big band.” Of course, the flute is loved by the public and is the most important classical instrument of the Symphony Wood Orchestra [7].

## 2. Related Work

The continuous development of digital technology and the continuous improvement of network technology have brought many cases to people's life. In addition to the initial communication, the current Internet technology has also played its role in many aspects of people's shopping and entertainment [8]. Moreover, the rich resources of the digitalization of the Internet provide people engaged in research and learning with important the supporting role [9]. Under the strategic idea of rejuvenating the country through science and education, online teaching in some relatively remote areas is an effective means. Literature pointed out that with the generalization of Internet digitalization and the maturity of algorithm programs, the current era of distance learning algorithm programs has become an irresistible development trend in the teaching field. This also puts forward high requirements for cultural training places such as universities, time, and crowd education mode [10]. There are many constraints for learners, and it is difficult to adapt to people's needs for knowledge. The continuous development of Internet technology has brought some development opportunities to traditional education and teaching, and online teaching has gradually developed. In the literature, distance learning tasks are used to transform the existing learning environment into the Internet, and uses new algorithms and new data sequences to ensure that distance education achieves better results than the old learning model [11, 12]. It is worth mentioning that the innovation of algorithm programs must express the scientific development concept of “people-oriented,” and the modern distance teaching model must be able to achieve “humanization” before it can develop into a truly humanized application mode [13]. The literature points out that with the wide use of algorithm programs in daily life, the introduction of algorithm programs into distance learning mode and the establishment of an automated Internet digital learning environment are becoming the mainstream trend of the future distance education system [14, 15].

### 2.1. Multi-Pass Scheduling Model and Algorithm Research of Separable Tasks

#### 2.1.1. Periodic Multi-Pass Scheduling Optimization Model for Separable Tasks with Given Scheduling Order

**Figure 1:**
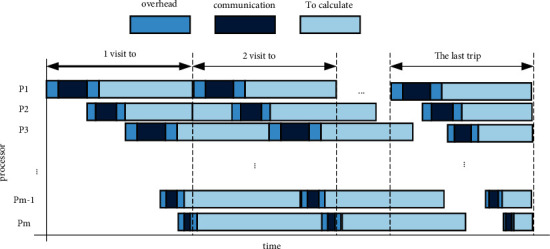
Multi-pass scheduling in blocking mode.

As shown in Figure 1, the layering of the Internet in the routing architecture determines that the mobile port of the Internet can also have a two-layer topology. One of them is the high-level self-determination management level department. They are considered the “real” topology, that is, the domain link rotation between the node in its own management department and the self-management department is constructed by domain links, and the other A topological structure is a low-level rotation level.

In general, in order to make the running algorithm time and the time of communicating information of one of the target machines the same as other machines, we have obtained the number of requirements of each machine from this. Therefore, you can get

(1) $$\begin{array}{c} \begin{array}{c} \alpha_{1}V\left(z_{1}+w_{1}\right)+o_{1}+s_{1}=\alpha_{2}V\left(z_{2}+w_{2}\right)+o_{2}+s_{2}\\ =\cdots \\ =\alpha_{m}V\left(z_{m}+w_{m}\right)+o_{m}+s_{m}, \end{array}\text{,} \end{array}$$

(2) $$\begin{array}{c} \alpha_{2}=\frac{\alpha_{1}V\left(z_{1}+w_{1}\right)+o_{1}+s_{1}-o_{2}-s_{2}}{V\left(z_{2}+w_{2}\right)}\text{.} \end{array}$$

It is known that

(3) $$\begin{array}{c} \alpha_{1}+\alpha_{2}+\cdots +\alpha_{m}=1\text{.} \end{array}$$

From (2) and (3), we can get

(4) $$\begin{array}{c} \alpha_{1}+\frac{\alpha_{1}V\left(z_{1}+w_{1}\right)+o_{1}+s_{1}-o_{2}-s_{2}}{V\left(z_{2}+w_{2}\right)}+\frac{\alpha_{1}V\left(z_{1}+w_{1}\right)+o_{1}+s_{1}-o_{3}-s_{3}}{V\left(z_{3}+w_{3}\right)}+\cdots +\frac{\alpha_{1}V\left(z_{1}+w_{1}\right)+o_{1}+s_{1}-o_{m}-s_{m}}{V\left(z_{m}+w_{m}\right)}=1\text{.} \end{array}$$

For the convenience of the following discussion, define the following two new variables:

(5) $$\begin{array}{c} \left\{\begin{array}{c} \Delta_{i}=\frac{z_{1}+w_{1}}{z_{i}+w_{i}}\text{,}\\ \Phi_{i}=\frac{o_{1}+s_{1}-o_{i}-s_{i}}{z_{i}+w_{i}}\text{.} \end{array}\right. \end{array}$$

Substituting (5) into simplified form:

(6) $$\begin{array}{c} \alpha_{1}\left(1+\overset{m}{\underset{i=2}{\sum }}\Delta_{i}\right)+\frac{1}{V}\overset{m}{\underset{i=2}{\sum }}\Phi_{i}=1\text{.} \end{array}$$

As a result, the optimal solution of the internal scheduling arrangement allocation plan can be obtained as shown in the following formula:

(7) $$\begin{array}{c} \left\{\begin{array}{c} \alpha_{1}=\frac{1-\left(1/V\right)\overset{m}{\underset{i=2}{\sum }}\Phi_{i}}{1+\overset{m}{\underset{i=2}{\sum }}\Delta_{i}}\text{,}\\ \alpha_{i}=\alpha_{1}\Delta_{i}+\frac{1}{V}\Phi_{i},i=1,2,\ldots m\text{.} \end{array}\right. \end{array}$$

Existing research indicates that in the final scheduling process, all slave processors must complete the algorithm formula together, so that it minimizes the total completion time of the task. Figure 1 shows a situation in which all processors in the system studied in this article complete job scheduling at one time, which allows the machine to have time to complete the job of the last trip of each slave processor, as shown in formulas (8) and (9).

(8) $$\begin{array}{c} T\left(\beta_{1}\right)=o_{1}+z_{1}\beta_{1}V+s_{1}+w_{1}\beta_{1}V\text{,} \end{array}$$

(9) $$\begin{array}{c} T\left(\beta_{i}\right)=\overset{i-1}{\underset{j=1}{\sum }}\left(o_{j}+z_{j}\beta_{j}V\right)+o_{i}+z_{i}\beta_{i}V+s_{i}+w_{i}\beta_{i}V\text{.} \end{array}$$

For all processors, the total completion time of the task is the same, namely,

(10) $$\begin{array}{c} T\left(\beta_{m}\right)=T\left(\beta_{m+1}\right)\text{.} \end{array}$$

Therefore,

(11) $$\begin{array}{c} s_{i}+w_{i}\beta_{i}V=o_{i+1}+z_{i+1}\beta_{i+1}V+s_{i+1}+w_{i+1}\beta_{i+1}V\text{,} \end{array}$$

(12) $$\begin{array}{c} \beta_{i+1}=\frac{s_{i}-\left(s_{i+1}+o_{i+1}\right)}{V\left(w_{i+1}+z_{i+1}\right)}+\frac{w_{i}}{w_{i+1}+z_{i+1}}\beta_{i}\text{.} \end{array}$$

For the sake of simplicity, two new variables are defined below:

(13) $$\begin{array}{c} \left\{\begin{array}{c} \delta_{i+1}=\frac{s_{i}-\left(s_{i+1}+o_{i+1}\right)}{w_{i+1}+z_{i+1}}\text{,}\\ \varepsilon_{i+1}=\frac{w_{i}}{w_{i+1}+z_{i+1}}\text{.} \end{array}\right. \end{array}$$

(11) can be simplified to

(14) $$\begin{array}{c} \beta_{i+1}=\frac{1}{V}\delta_{i+1}+\varepsilon_{i+1}\beta_{i}\text{.} \end{array}$$

Let us set it up:

(15) $$\begin{array}{c} \delta_{i}\geq 0\text{.} \end{array}$$

If the workload of calculation is very heavy, let all those who can work participate in the calculation operation; then, the assumption is correct, and if *β*_*i*_ is negative, then Pi does not participate in the calculation. So, to sum up, (13) can be changed to

(16) $$\begin{array}{c} \left\{\begin{array}{c} E_{i}=\overset{i}{\underset{j=2}{\prod }}\varepsilon_{j},\\ \Gamma_{i}=\overset{i}{\underset{j=2}{\sum }}\left(\delta_{j}\overset{i}{\underset{k=j+1}{\prod }}\varepsilon_{k}\right)\text{.} \end{array}\right. \end{array}$$

Because

(17) $$\begin{array}{c} \sum_{i=1}^{m}\beta_{i}=1 \end{array}$$

then

(18) $$\begin{array}{c} \left\{\begin{array}{c} \beta_{1}=\frac{1-1V\overset{m}{\underset{i=2}{\sum }}\Gamma_{i}}{1+\overset{m}{\underset{i=2}{\sum }}E_{i}}\text{,}\\ \beta_{2}=E_{i}\beta_{1}+\frac{1}{V}\Gamma_{i}i=2,\ldots ,m\text{.} \end{array}\right. \end{array}$$

After formula (18), in systems with different structures, it is easy to obtain the number of tasks assigned to each slave processor in the final adjustment process, where the cost of starting information transmission and the starting cost of algorithm operation should be considered.

Assuming that the number of slave processors participating in the algorithm operation is *m* and the number of reserved strokes is *n* + 1, then a separable task optimization scheduling model can be obtained. The following is a description of the sample.

(19) $$\begin{array}{c} \min_{n,m}T\left(W\right)=\min\;\left[n\left(\alpha_{1}V\left(z_{1}+w_{1}\right)+o_{1}+s_{1}\right)+\beta V\left(z_{1}+w_{1}\right)+o_{1}+s_{1}\right]\text{,}\\ subjecte\;d\;to\\ \left\{\begin{array}{c} \alpha_{i}>0,i\in \left\{1,2,\ldots ,m\right\},\\ \overset{m}{\underset{i=1}{\sum }}\alpha_{i}=1,\\ \beta_{i}>0,i\in \left\{1,2,\ldots ,m\right\},\\ \overset{m}{\underset{i=1}{\sum }}\beta_{i}=1. \end{array}\right. \end{array}$$

### 2.2. Verification of Inspection Results for a Given Scheduling Sequence

In the problem of enhanced knowledge, the terminal artificially implanted in the intelligent program usually does not know the model of the environment and can only update itself by constant trial and error. The traditional enhanced knowledge algorithm often has unstable results in the complex MDP environment. There is no guarantee that it will converge to the optimal strategy every time. There are many reasons for this result. For example, the surrounding environment is too chaotic. The terminal artificially implanted in the intelligent program cannot fully judge the surrounding situation. The algorithm of the artificially implanted intelligent program terminal itself is flawed, and the environment estimation is inaccurate, resulting in errors and instability. Since the sample algorithm discussed in this chapter is for the samples proposed in the material, there are many articles in the master's thesis of Xidian University in Wanfang's library. This experiment will be studied, and a new algorithm will be proposed as a comparison algorithm, to compare it with the FPMISA proposed in this experiment. The main reason for random variance is the use of algorithms with random equations, such as *ϵ*-greedy algorithm, when updating the value equation. The classical reinforcement knowledge algorithm Sarsa and *Q*-learning algorithm both use the *ϵ*-greedy algorithm in the action strategy, but in the update strategy, the greedy algorithm used by Q-learning and the *ϵ*-greedy algorithm used by Sarsa are updated by changing the *ϵ*-greedy selection. It becomes the expectation equation, which effectively reduces the random variance of the original Sarsa at the cost of increasing the complexity of the algorithm. The number of processor parameters is shown in Table 1.

In the literature, a kind of command algorithm that can be allocated in a heterogeneous distributed system is proposed to find multiple positive solutions. In the following experiments, the algorithm proposed in this section will be used in the range of 50,000 to 40,000 tasks. FPMISA and Amin's method are performed under the same scheduling order. Schedule and compare results. The two scheduling sequences used in the experiment are represented by IZ in the order of increasing information transmission rate and IW in the order of increasing algorithm running rate. It can be seen from Table 2 that under a large amount of tasks, whether it is scheduling using sequential IZ or IW, the proposed algorithm has the following two characteristics: first, it uses more processors, and second it uses less scheduling. In terms of the number of passes, it can be seen that the algorithms proposed in this article have achieved relatively good scheduling results. For the experimental results, the analysis is as follows. The algorithm proposed in this article gives priority to using more processors under the premise of producing feasible solutions, which can make better and more full use of the system's algorithm operation resources and improve the concurrency of the system. At the same time, the running program of this article can solve the most suitable number of scheduling trips. While reducing the idle time between processors, it will not introduce too many scheduling trips, thus avoiding excessive startup overhead. In summary, the new algorithm proposed in this article can solve better scheduling results more efficiently. Table 2 shows the task completion time comparison test.

### 2.3. Dividable Task Regularity Multiple Scheduling Model of Optimal Scheduling Order

Same as the explanation in the previous section, it derives a new regular multi-pass scheduling model for separable tasks to solve the optimal scheduling sequence.

**Figure 2:**
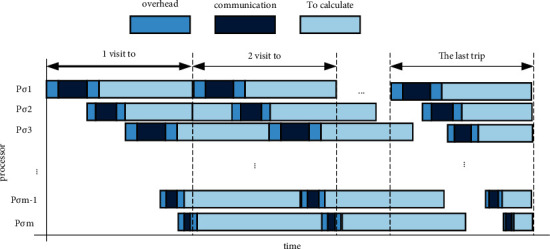
Multiple scheduling with obstructive mode considering scheduling order.

As shown in Figure 2, in each internal scheduling, in order to make a slave processor such as P complete its current algorithm, the central processing system can give it the next instruction that needs to be operated. Therefore, for all processing, we strive to make the information transmission and algorithm time of each processor the same for each internal scheduling. In short, we have determined the number of instructions for each processor so that the information transmission and algorithm running time of each processor is equal to the information transmission and algorithm running time of all other processors. From this, we can get

(20) $$\begin{array}{c} \begin{array}{c} \alpha_{\sigma_{1}}V\left(z_{\sigma_{1}}+w_{\sigma_{1}}\right)+o_{\sigma_{1}}+s_{\sigma_{1}}=\alpha_{\sigma_{2}}V\left(z_{\sigma_{2}}+w_{\sigma_{2}}\right)+o_{\sigma_{2}}+s_{\sigma_{2}}\\ =\ldots \\ =\alpha_{\sigma_{m}}V\left(z_{\sigma_{m}}+w_{\sigma_{m}}\right)+o_{\sigma_{m}}+s_{\sigma_{m}} \end{array}\text{,} \end{array}$$

(21) $$\begin{array}{c} \alpha_{\sigma_{2}}=\frac{\alpha_{\sigma_{1}}V\left(z_{\sigma_{1}}+w_{\sigma_{1}}\right)+o_{\sigma_{1}}+s_{\sigma_{1}}-o_{\sigma_{2}}-s_{\sigma_{2}}}{V\left(z_{\sigma_{2}}+w_{\sigma_{2}}\right)}\text{,} \end{array}$$

(22) $$\begin{array}{c} \alpha_{\sigma_{1}}+\alpha_{\sigma_{2}}+\cdots +\alpha_{\sigma_{m}}=1\text{.} \end{array}$$

From (21) and (22), we can get

(23) $$\begin{array}{c} \begin{array}{c} \alpha_{\sigma_{1}}+\frac{\alpha_{\sigma_{1}}V\left(z_{\sigma_{1}}+w_{\sigma_{1}}\right)+o_{\sigma_{1}}+s_{\sigma_{1}}-o_{\sigma_{2}}-s_{\sigma_{2}}}{V\left(z_{\sigma_{2}}+w_{\sigma_{2}}\right)}\\ +\frac{\alpha_{\sigma_{1}}V\left(z_{\sigma_{1}}+w_{\sigma_{1}}\right)+o_{\sigma_{1}}+s_{\sigma_{1}}-o_{\sigma_{3}}-s_{\sigma_{3}}}{V\left(z_{\sigma_{3}}+w_{\sigma_{3}}\right)}\\ +\ldots \end{array}\text{,} \end{array}$$

(24) $$\begin{array}{c} +\frac{\alpha_{\sigma_{1}}V\left(z_{\sigma_{1}}+w_{\sigma_{1}}\right)+o_{\sigma_{1}}+s_{\sigma_{1}}-o_{\sigma_{m}}-s_{\sigma_{m}}}{V\left(z_{\sigma_{m}}+w_{\sigma_{m}}\right)}=1\text{.} \end{array}$$

For the convenience of the following discussion, define the following two new variables:

(25) $$\begin{array}{c} \left\{\begin{array}{c} \Delta_{\sigma_{i}}=\frac{z_{\sigma_{1}}+w_{\sigma_{1}}}{z_{\sigma_{i}}+w_{\sigma_{i}}}\text{,}\\ \Phi_{\sigma_{i}}=\frac{o_{\sigma_{1}}+s_{\sigma_{1}}-o_{\sigma_{i}}-s_{\sigma_{i}}}{z_{\sigma_{i}}+w_{\sigma_{i}}}\text{.} \end{array}\right. \end{array}$$

Substituting (22) into simplified form:

(26) $$\begin{array}{c} \alpha_{\sigma_{i}}\left(1+\overset{m}{\underset{i=2}{\sum }}\Delta_{\sigma_{i}}\right)+\frac{1}{V}\overset{m}{\underset{i=2}{\sum }}\Phi_{\sigma_{i}}=1\text{.} \end{array}$$

The results show that the compact solution of the internal scheduling task allocation strategy can be obtained as

(27) $$\begin{array}{c} \left\{\begin{array}{c} \alpha_{\sigma_{i}}=\frac{1-\left(1/V\right)\overset{m}{\underset{i=2}{\sum }}\Phi_{\sigma_{i}}}{1+\overset{m}{\underset{i=2}{\sum }}\Delta_{\sigma_{i}}}\text{,}\\ \alpha_{\sigma_{i}}=\alpha_{\sigma_{1}}\Delta_{\sigma_{i}}+\frac{1}{V}\Phi_{\sigma_{i}},i=1,2,\ldots m\text{.} \end{array}\right. \end{array}$$

Research shows that when scheduling, the proposed algorithm has the following two characteristics: first, it uses more processors, and second, it uses less scheduling. It can be seen that the algorithms proposed in this article have achieved relatively better scheduling results. For the experimental results, the analysis is as follows. The algorithm proposed in this article will give priority to using more processors on the premise that it produces feasible solutions, which can make better use of the system's algorithm running resources and improve the concurrency of the system. Therefore, the task completion time of the last pass of any processor can be obtained, as shown below.

(28) $$\begin{array}{c} T\left(\beta_{\sigma_{i}}\right)=o_{\sigma_{i}}+z_{\sigma_{i}}\beta_{\sigma_{i}}V+s_{\sigma_{i}}+w_{\sigma_{i}}\beta_{\sigma_{i}}V\text{,}\\ T\left(\beta_{\sigma_{i}}\right)=\overset{i-1}{\underset{j=1}{\sum }}\left(o_{j}+z_{\sigma_{j}}\beta_{\sigma_{j}}V\right)+o_{\sigma_{i}}+z_{\sigma_{i}}\beta_{\sigma_{i}}V+s_{\sigma_{i}}+w_{\sigma_{i}}\beta_{\sigma_{i}}V\text{.} \end{array}$$

It can be known that for each processor, the time to complete the task is always the same, which is

(29) $$\begin{array}{c} T\left(\beta_{\sigma_{m}}\right)=T\left(\beta_{\sigma_{m+1}}\right)\text{.} \end{array}$$

So, we have

(30) $$\begin{array}{c} s_{\sigma_{i}}+w_{\sigma_{i}}\beta_{\sigma_{i}}V=o_{\sigma_{i+1}}+z_{\sigma_{i+1}}\beta_{\sigma_{i+1}}V+s_{\sigma_{i+1}}+w_{\sigma_{i+1}}\beta_{\sigma_{i+1}}V\text{,} \end{array}$$

(31) $$\begin{array}{c} \beta_{\sigma_{i+1}}=\frac{s_{\sigma_{i}}-\left(s_{\sigma_{i+1}}+o_{\sigma_{i+1}}\right)}{V\left(w_{\sigma_{i+1}}+z_{\sigma_{i+1}}\right)}+\frac{w_{\sigma_{i}}}{w_{\sigma_{i+1}}+z_{\sigma_{i+1}}}\beta_{\sigma_{i}}\text{.} \end{array}$$

For the convenience of discussion, two new variables are defined below:

(32) $$\begin{array}{c} \left\{\begin{array}{c} \delta_{\sigma_{i+1}}=\frac{s_{\sigma_{i}}-\left(s_{\sigma_{i+1}}+o_{\sigma_{i+1}}\right)}{w_{\sigma_{i+1}}+z_{\sigma_{i+1}}}\text{,}\\ \varepsilon_{\sigma_{i+1}}=\frac{w_{\sigma_{i}}}{w_{\sigma_{i+1}}+z_{\sigma_{i+1}}}\text{.} \end{array}\right. \end{array}$$

(31) can be simplified to

(33) $$\begin{array}{c} \beta_{\sigma_{i+1}}=\frac{1}{V}\delta_{\sigma_{i+1}}+\varepsilon_{\sigma_{i+1}}\beta_{\sigma_{i}}\text{.} \end{array}$$

In which

(34) $$\begin{array}{c} \delta_{\sigma_{i}}\geq 0\text{.} \end{array}$$

When there are many targets, let all processors join the operation; then, this conjecture can be established, and if *β*_*σ*_*i*__ is negative, it is equivalent to *P*_*σ*_*i*__ not participating in scheduling. After running with the program algorithm, (34) can be transformed into

(35) $$\begin{array}{c} \left\{\begin{array}{c} E_{\sigma_{i}}=\overset{i}{\underset{j=2}{\prod }}\varepsilon_{\sigma_{j}}\text{,}\\ \Gamma_{\sigma_{i}}=\overset{i}{\underset{j=2}{\sum }}\left(\delta_{\sigma_{j}}\overset{i}{\underset{k=j+1}{\prod }}\varepsilon_{\sigma_{k}}\right)\text{.} \end{array}\right. \end{array}$$

Because

(36) $$\begin{array}{c} \sum_{i=1}^{m}\beta_{\sigma_{i}}=1\text{,} \end{array}$$

then

(37) $$\begin{array}{c} \left\{\begin{array}{c} \beta_{\sigma_{1}}=\frac{1-1V\overset{m}{\underset{i=2}{\sum }}\Gamma_{\sigma_{i}}}{1+\overset{m}{\underset{i=2}{\sum }}E_{\sigma_{i}}}\text{,}\\ \beta_{\sigma_{2}}=E_{\sigma_{i}}\beta_{\sigma_{1}}+\frac{1}{V}\Gamma_{\sigma_{i}}i=2,\ldots ,m\text{.} \end{array}\right. \end{array}$$

From the calculation of (35), considering the influence of the initial cost of information transmission and the initial cost of running algorithms in a heterogeneous system, the workload allocated to each slave processor in the last scheduling can be easily obtained.

It is conjectured that the number of subordinate processors participating in the algorithm operation is *m* and the number of scheduling passes is *n* 1, where *n* is the number of structural scheduling, so that a scheduling sample with separable tasks can be constructed. The situation of the model is as follows:

(38) $$\begin{array}{c} \min_{n,m}T\left(W\right)=\min\;\left[n\left(\alpha_{\sigma_{1}}V\left(z_{\sigma_{1}}+w_{\sigma_{1}}\right)+o_{\sigma_{1}}+s_{\sigma_{1}}\right)+\beta V\left(z_{\sigma_{1}}+w_{\sigma_{1}}\right)+o_{\sigma_{1}}+s_{\sigma_{1}}\right]\text{,}\\ subjecte\;d\;to\\ \left\{\begin{array}{c} \alpha_{\sigma_{i}}>0,i\in \left\{1,2,\ldots ,m\right\},\\ \overset{m}{\underset{i=1}{\sum }}\alpha_{\sigma_{i}}=1,\\ \beta_{\sigma_{i}}>0,i\in \left\{1,2,\ldots ,m\right\},\\ \overset{m}{\underset{i=1}{\sum }}\beta_{\sigma_{i}}=1. \end{array}\right. \end{array}$$

### 2.4. Analysis of the Experimental Results of the Optimal Scheduling Sequence

This section will explain a brand-new scheduling order of multiple scheduling samples of regular assignment tasks. In reinforcement learning, the variance of the estimated value is also an important factor affecting its stability. Even if the expected value of the estimated value is relatively accurate, if the variance is too high, severe swings in the estimated value will make the strategy swing with it and ultimately lead to unstable results. Also, for the model manufacturing, a new comprehensive improved genetic algorithm is proposed for calculation.

The relevant parameter indicators of the machine in this experiment are as follows. In the experiment, the algorithm FPMISA and Amin's method proposed in this section will be used in the range of 50,000 to 40,000 tasks to perform scheduling and compare the results in the same scheduling order. The two scheduling sequences used in the experiment are represented by IZ in the order of increasing information transmission rate and IW in the order of increasing algorithm running rate. Under a larger task load, whether it is to use sequential IZ or IW for scheduling, the proposed algorithm has the following two characteristics: first, it uses more processors, and second, it uses fewer scheduling passes. The algorithms proposed in this article have achieved relatively good scheduling results. For the experimental results, the analysis is as follows. The algorithm proposed in this article gives priority to using more processors under the premise of producing feasible solutions, which can make better and more full use of the system's algorithm operation resources and improve the concurrency of the system.  Table 3 shows the experimental parameters.

The relevant letter settings below are expressed in the experiment on genetic algorithms as follows: the total sample size PopSize 100, the interleaving probability *p*_*c*_=0.6, the change probability *p*_*m*_=0.02, the maximum number of replacements is 2000 generations, and the number of high-level individuals is 5.

In the literature, the scheduling order proposed by Hsu is *z*_*i*_/(*z*_*i*_ + *w*_*i*_), which is a gradually increasing order; the set is *m*, *n* + 1 and the total completion time is under the range of 50,000 to 400,000 in total.  Table 4 shows the task completion time comparison experiment.

The experimental results in Table 5 show the optimal scheduling order obtained by the algorithm of this article under different tasks. It can be seen from the table that with the difference in the amount of tasks, the optimal scheduling order obtained by the solution has also changed. On the other hand, it shows that there is no fixed scheduling order that can achieve the best under different tasks. Optimal scheduling results demonstrated that the scheduling sequence has an important impact on the task scheduling results.

## 3. Design and Application of Flute Music Remote Teaching System for Artificial Intelligence

### 3.1. System Structure Design

**Figure 3:**
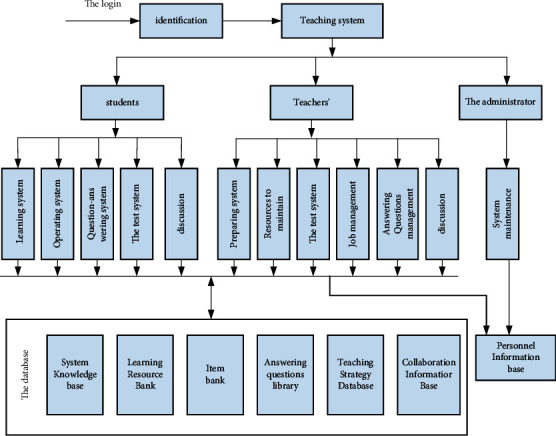
Function diagram of distance teaching system.

For a relatively complete teaching system with distance education mode functions, the following main supporting functional sections are included, such as online learning section, online course preparation module, homework module, examination module, question and answer module, information discussion module, and information processing module. As shown in Figure 3, these functional modules are independent of each other and communicate through the system. Through the interconnection between the data, the data can be systematized and an effective support can be constructed.

**Figure 4:**
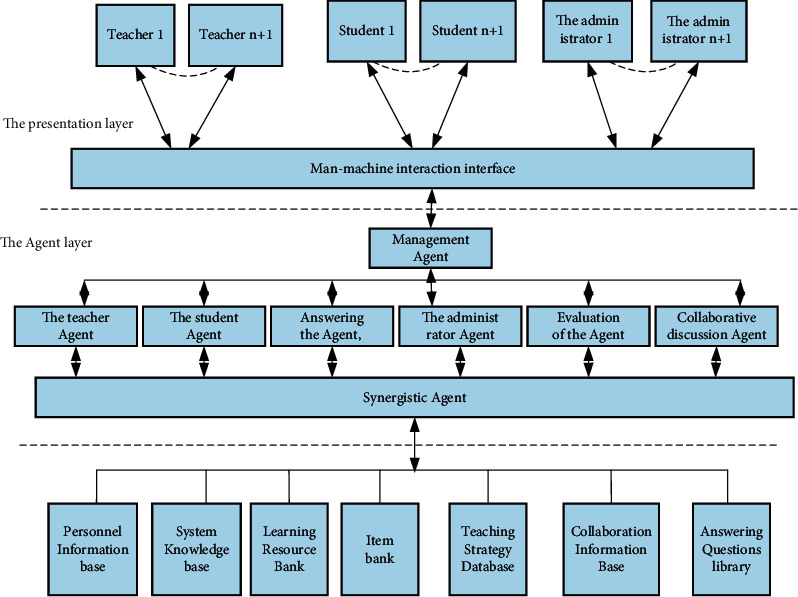
Targeted distance teaching system model.

Each distance teaching platform in our country is in its own line, with different positioning and different teaching content. For Internet teaching, self-acquisition of knowledge is the core. Therefore, in the process of teaching, teachers should conduct individual students' autonomous learning awareness. Strengthen it to form the habit and ability of independent learning. At the same time, we should also advocate the integration of “teaching” and “learning,” provide timely test feedback to students, consider students, and conduct one-to-one instructional learning for them. These suggestions are for data system reasoning. Both have great reference value. This information includes the user's visit records, the completion of user operations and test data, the discussion and exchange of online participation, the user's interests and specialties and operation methods, learning progress and scoring, and so on. Most of the system is divided into a number of different areas for identification, and these areas will deal with the various scenes encountered in teaching. According to the division of these functional sections, we can create the level of help to solve the problem and build by these to help users complete the required tasks. Each functional module has mutual dependence, which can be solved through agent interaction and cooperation. Through the above analysis and explanation, we have established many samples of modern distance education systems to solve the above difficulties. Its schematic diagram is shown in Figure 4.

From the above schematic diagram, it can be found that the entire teaching system is divided into three levels in a logical sense:

1. Basic database layer: the database system that stores all the data in the system.
2. Management agent: it is used to help various learning, which contains 8 components, namely, agent student, agent, evaluation agent, agent Q&A, management teacher agent, administrator agent, collaborative discussion agent, and collaborative agent.
3. Interface presentation layer: it is the interface between the user and the system, including the interactive man-machine interface. It mainly provides assistance to three types of users: instructors, researchers, and managers.

### 3.2. Construction of Each Agent in the System

The construction of agent is the most time-consuming and most difficult work in the entire system. If you start research and construction directly at the bottom, it takes too much time and energy, and it is not easy to put more energy into the realization of functions and ideas. Therefore, building a mature service sector is the most effective and wise choice, and the platform can also quickly build multiple sectors. The platform invented a simple configuration method, so users do not need to pay attention to the transmission of basic information and structure. Since it is developed for the platform, the interface is also very easy to use.

This information includes the user's visit records, the completion of user operations and test data, the discussion and exchange of online participation, the user's interests and specialties and operation methods, learning progress and scoring, and so on. Moreover, they are clearly stratified samples. These factors allow users to carry out relevant research according to their different needs and different levels. Because the level of agent intelligence of this platform is not perfect, it is necessary to rely on the methods created by the developers to achieve its intelligence requirements. Most of the system is divided into a number of different areas for identification, and these areas will deal with the various scenes encountered in teaching. According to the division of these functional sections, we can create the level of help to solve the problem and build by these to help users complete the required tasks. Each functional module has mutual dependence, which can be solved through agent interaction and cooperation. Through the above analysis and explanation, we have established many samples of modern distance education systems to solve the above difficulties. The ability to create an agent construction model efficiently and quickly puts forward higher requirements on the inventors, which can only be achieved in thought with extra effort and time.

**Figure 5:**
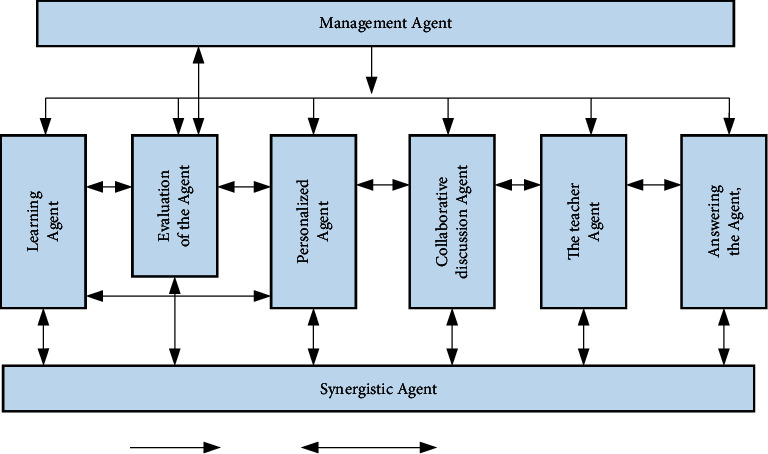
Agent relationship diagram.

The management agent in the system is used to help various learning, which contains 8 components, namely, student agent, agent, evaluation agent, agent Q&A, management teacher agent, administrator agent, collaborative discussion agent, and collaborative agent. The KQML language is used to transfer information between agents, so as to exchange information to complete tasks. The way to help learners complete the research tasks is to complete the task by sharing tasks. The specific levels between the agents are shown in Figure 5.

### 3.3. System Development and Testing

At present, many applications of Internet digitization are aimed at the system development tool B/S browser server mode. The model mentioned in this article is also aimed at the B/S model, which uses the JPS algorithm program to express the development platform of the application system and uses this as an innovative development tool for the database. Students choose the courses they need to study according to the study plan to start learning. When a student enters the system, the algorithm program will automatically create a personalized code corresponding to the student. At the same time, the program can also track whether the student has reached the teaching standard. The homework in the course is a way for students to practice the learning outcomes in class and deepen their understanding of the learning outcomes. After completing a specific knowledge point, it is necessary to repeatedly consolidate the foundation. Students only need to click the “class practice” button, and the database will call the exercises of the relevant task points in the practice library. When the student completes the question and clicks the “Submit Assignment” button, the system will submit the data and compare the provided test with the correct answer to score, and the score of the question made by the student is displayed to the student. For the wrong question, the system gives correct answers for students' reference. When the students get low scores in the practice of a certain knowledge point, the system will automatically process the data, and the user will not provide the answering process to the related questions, so that the students can learn again.

The system will store the learning situation of each student in a database of individual student information and use it as a sample to guide future students. As a complete education model, the test system is an essential part. Testing is the most direct and effective way to test students' learning effects. The test link can enable teachers to have a clear understanding of students' knowledge acquisition and can comprehensively evaluate students through their learning achievements and daily performance. At the same time, it also gives the next learning strategy according to the student's learning effect, extracts the reasonable results, finds some related problems, and puts the information in the hands of the students. If you cannot find any useful knowledge or your students have objections to the answers found, ask the teacher a question, and the system will reflect the answer to the students. When the student closes the program and clicks the “Logout” button after completing the current study task, it may also interrupt the research process due to abnormalities and unexpected circumstances, and it will also save the information recorded by the student to its database. At the same time, it manages the abnormal state of students to repair and open resources.

### 3.4. Suggestions for Flute Music Teaching and Development

If you want to endure for a long time and pass on an art form from generation to generation, it must show its artistic value, and it must be handed down to pass it on. The same is true for flute art. Ever since the flute was invented in the West, classic achievements have been made. The Internet in the 21st century has brought many opportunities for development, and the Internet has brought another “spring” to flute art. Therefore, adding new technology to the existing flute music algorithm program is another opportunity to reform the flute art. Teenagers are the infinite motive force for social development. Therefore, it is necessary to do a good job in enlightening children's flute education to lay a solid foundation for training specialized flute talents in the future. The author believes that forming a school band is a very good idea. Children must establish a sense of cooperation with others in the band, learn from each other, and develop in a common environment. Modern children's flute enlightenment education is different from previous education. The former flute classroom model was a 1:1 classroom with a teacher and a student, but now it is a group classroom model where a group of 3 to 5 people study and research together. The hot topic of discussion is whether to practice together or compete together. This way of competing with each other will cultivate children's enthusiasm. Today's society is no longer a society that advocates individual efforts but is a win-win society. Therefore, the ideological enlightenment education of juvenile flute must keep up with the development and progress of society.

In the long-distance flute teaching in our country, the relatively backward technical equipment in our country is the evaluation system and monitoring. These two factors are the main culprits for students' inability to learn musical instruments efficiently. Through the establishment of a certain foundation of constant supervision and network problem diagnosis and through tests, mock examinations, and issuance of certificates, domestic students can easily learn through distance education. There are many ways of supervision, which can be divided into final evaluation and self-scoring. There are many types of detection methods, using the positioning system to track and store learners' learning information. In the remote communication, the acceptance of students' activities or learning results is of great help to students, which is more effective than one-to-one effects. Although everyone's understanding and level of music are different, flute happens to be an art form that has no hard indicators to evaluate its quality. In the class mode of music schools at home and abroad, a large number of large-scale lectures are used and the commonality of flute is discussed together, while the short-term courses focus on the execution of algorithm programs. This can not only effectively enhance students' cognition of their own performance level but also broaden students' understanding and ideas.

## 4. Conclusion

The development of Internet technology has brought convenience to people's life, so the traditional teaching methods are bound to produce some changes. Intellectuals must first have certain “information processing ability” and “novel creative ability.” However, the current old Internet digital teaching model has many problems, such as low intelligence, weak interaction, lack of effective learning guidance mechanism, lack of effective evaluation mechanism, and so on, which makes it difficult to satisfy people's needs in Internet digital teaching. There is need for knowledge. This article is aimed at the problems encountered by the old Internet digital education, such as the low level of artificial intelligence, the lack of information processing ability, the lack of effective learning ability, the lack of mature self-improvement ability, and so on. The thirst for knowledge can hardly be satisfied by the current Internet digital teaching. We propose a new algorithm program in the field of reasonable allocation of artificial intelligence: the invention of the agent algorithm program is an Internet digital teaching system for algorithm programs. This article has carried out a comprehensive and systematic explanation and analysis of the agent algorithm program. In addition, it explores and explains all the key steps required by the current distance education model and invents a sample of the distance education model. This article preliminarily summarizes the automatic teaching and personalized education functions of this model, laying the foundation for future development and exploration in this field.

## Figures and Tables

**Table 1:** Experimental parameters.

*P*	o	s	z	w
P_1_	4.11	1.59	0.44	6.82
P_2_	10.23	2.98	0.67	1223
P_3_	17.04	4.28	0.95	18.48
P_4_	11.58	3.12	0.76	13.84
P_5_	6.29	1.91	0.59	8.90
P_6_	11.67	3.56	0.78	13.53
P_7_	17.25	5.47	0.96	19.04
P_8_	9.43	3.74	0.53	27.05
P_9_	1.43	1.99	0.16	8.06
P_10_	4.32	2.13	0.42	9.96
P_11_	7.50	2.57	0.64	11.90
P_12_	5.38	2.43	0.44	12.42
P_13_	3.37	2.45	0.25	13.80
P_14_	2.98	3.06	0.24	14.84
P_15_	2.28	3.60	0.23	15.64
P_16_	6.56	2.43	0.52	10.62
P_17_	10.78	1.22	0.82	5.78
P_18_	10.34	2.51	0.78	8.74
P_19_	11.90	3.88	0.83	11.92
P_20_	10.08	2.70	0.72	8.84
P_21_	8.13	1.52	0.63	5.62
P_22_	5.32	3.28	0.81	10.28
P_23_	2.50	5.06	0.18	16.12
P_24_	1.86	3.98	0.16	12.03
P_25_	1.69	2.92	0.14	9.36
P_26_	5.42	2.78	0.46	9.47
P_27_	8.02	2.60	0.79	9.68
P_28_	3.78	3.52	0.44	11.19
P_29_	2.57	4.26	0.19	13.48
P_30_	6.38	3.78	0.37	15.39

**Table 2:** Task completion time comparison test.

Algorithm	Task volume	Scheduling sequence	*m* + 1	m	Complete time
Amin's method FPMISA	50000	LZ	3	21	30136
		LW	89	12	37346
		LZ	3	30	23098
		LW	42	30	19912

Amin's method FPMISA	100000	LZ	7	22	54900
		LW	123	13	69650
		LZ	6	30	42218
		LW	60	30	39338

Amin's method FPMISA	150000	LZ	10	22	80938
		LW	1487	14	98388
		LZ	9	30	61339
		LW	73	30	53636

Amin's method FPMISA	200000	LZ	12	23	103279
		LW	170	14	130899
		LZ	12	30	80459
		LW	84	30	77994

Amin's method FPMISA	250000	LZ	17	23	128413
		LW	190	14	163379
		LZ	15	30	995800
		LW	94	30	97276

Amin's method FPMISA	300000	LZ	21	23	153418
		LW	208	14	195873
		LZ	18	30	118700
		LW	103	30	116540

Amin's method FPMISA	350000	LZ	25	23	178445
		LW	222	15	217231
		LZ	21	30	137821
		LW	112	30	135790

Amin's method FPMISA	400000	LZ	28	22	203557
		LW	238	15	248088
		LZ	25	30	156815
		LW	119	30	155029

**Table 3:** Experimental parameters.

P	o	s	z	w
P_1_	4.11	1.59	0.44	6.82
P_2_	10.23	2.98	0.67	12.23
P_3_	17.04	4.28	0.95	18.48
P_4_	11.58	3.12	0.76	13.84
P_5_	6.29	1.91	0.59	8.90
P_6_	11.67	3.56	0.78	13.53
P_7_	17.25	5.47	0.96	19.04
P_8_	9.43	3.74	0.53	27.05
P_9_	1.43	1.99	0.16	8.06
P_10_	4.32	2.13	0.42	9.96
P_11_	7.50	2.57	0.64	11.90
P_12_	5.38	2.43	0.44	12.42
P_13_	3.37	2.45	0.25	13.80
P_14_	2.98	3.06	0.24	14.84
P_15_	2.28	3.60	0.23	15.64
P_16_	6.56	2.43	0.52	10.62
P_17_	10.78	1.22	0.82	5.78
P_18_	10.34	2.51	0.78	8.74
P_19_	11.90	3.88	0.83	11.92
P_20_	10.08	2.70	0.72	8.84
P_21_	8.13	1.52	0.63	5.62
P_22_	5.32	3.28	0.81	10.28
P_23_	2.50	5.06	0.18	16.12
P_24_	1.86	3.98	0.16	12.03
P_25_	1.69	2.92	0.14	9.36
P_26_	5.42	2.78	0.46	9.47
P_27_	8.02	2.60	0.79	9.68
P_28_	3.78	3.52	0.44	11.19
P_29_	2.57	4.26	0.19	13.48
P_30_	6.38	3.78	0.57	15.39

**Table 4:** Task completion time comparison experiment.

Algorithm	Task volume	*n* + 1	m	Complete time
IZ	50000	3	21	30186
IW		89	12	37346
Hsu		37	22	29554
GA		33	30	19725

IZ	100000	7	22	54900
IW		123	13	69650
Hsu		56	23	55404
GA		48	30	39097

IZ	150000	10	22	80985
IW		147	14	98388
Hsu		63	24	79507
GA		57	30	58370

IZ	200000	14	23	103279
IW		170	14	130899
Hsu		72	24	105806
GA		66	30	77630

IZ	250000	17	23	128413
IW		190	14	163379
Hsu		81	24	132091
GA		74	30	96870

IZ	300000	21	23	153418
IW		208	14	195837
Hsu		89	24	158362
GA		81	30	116095

IZ	350000	25	23	178445
IW		222	15	217231
Hsu		96	24	184622
GA		87	30	135310

IZ	400000	28	23	203557
IW		238	15	248088
Hsu		103	24	210876
GA		93	30	154519

**Table 5:** The optimal scheduling sequence under different target quantities.

Task volume	Scheduling sequence
50000	23, 9, 25, 24, 29, 1, 13, 14, 15, 10, 28, 12, 26, 16, 8, 30, 11, 2, 5, 4, 20, 6, 18, 27, 22, 19, 21, 17, 3, 7
100000	25, 9, 24, 1, 15, 21, 23, 29, 14, 13, 10, 28, 12, 26, 16, 8, 30, 5, 11, 2, 20, 4, 6, 18, 27, 22, 19, 17, 3, 7
150000	24, 9, 25, 29, 15, 23, 1, 13, 14, 10, 28, 12, 26, 16, 8, 30, 11.5, 2, 4, 20, 6, 18, 27, 22, 19, 21, 17, 3, 7
200000	24, 9, 25, 29, 14, 23, 1, 13, 15, 10, 28, 12, 26, 16, 30, 5, 8, 11, 2, 20, 4, 6, 18, 27, 22, 19, 21, 17, 3, 7
250000	23, 9, 25, 24, 29, 15.1, 14, 13, 10, 12, 28, 26, 30, 16, 8, 5, 11, 2, 20, 4, 6, 27, 18, 22, 21, 17, 19, 7, 3
300000	23, 9, 25, 24, 29, 14, 1, 15, 13, 10, 28, 12, 26, 16, 8, 30, 5, 11, 2, 20, 4.18, 6, 27, 22, 21, 17, 19, 3, 7
350000	23, 9, 25, 24, 29, 15, 1, 14, 13, 10, 28, 12, 26, 16, 8, 30, 5, 11, 2, 20, 4, 18, 6, 27, 22, 21, 17, 19, 3, 7
400000	24, 9, 25, 29, 13, 15, 23, 1, 14, 10, 28, 12, 26, 16, 8, 30, 5, 11, 2, 20, 4, 18, 6, 27, 22, 21, 19, 17, 3, 7

## Data Availability

The data used to support the findings of this study are available from the corresponding author upon request.
